# Monoexponential, biexponential and diffusion kurtosis MR imaging models: quantitative biomarkers in the diagnosis of placenta accreta spectrum disorders

**DOI:** 10.1186/s12884-022-04644-9

**Published:** 2022-04-22

**Authors:** Tao Lu, Yishuang Wang, Aiwen Guo, Wei Cui, Yazheng Chen, Shaoyu Wang, Guotai Wang

**Affiliations:** 1 Department of Radiology, Sichuan Academy of Medical Sciences & Sichuan Provincial People’s Hospital, 32 West Second Section, First Ring Road, Chengdu, 610072 China; 2 Siemens Healthineer, No.278, Zhouzhu Road, Pudong New Area District, Shanghai, 201318 China; 3 School of Mechanical and Electrical Engineering, University of Electronic Science and Technology of China, 2006 Xiyuan Avenue, West Hi-tech Zone, Chengdu, 611731 China

**Keywords:** PAS disorders, Diffusion-weighted MRI, Intravoxel incoherent motion, Diffusion kurtosis imaging

## Abstract

**Background:**

To investigate the diagnostic value of monoexponential, biexponential, and diffusion kurtosis MR imaging (MRI) in differentiating placenta accreta spectrum (PAS) disorders.

**Methods:**

A total of 65 patients with PAS disorders and 27 patients with normal placentas undergoing conventional DWI, IVIM, and DKI were retrospectively reviewed. The mean, minimum, and maximum parameters including the apparent diffusion coefficient (ADC) and exponential ADC (eADC) from standard DWI, diffusion kurtosis (MK), and mean diffusion coefficient (MD) from DKI and pure diffusion coefficient (D), pseudo-diffusion coefficient (D*), and perfusion fraction (f) from IVIM were measured from the volumetric analysis and compared between patients with PAS disorders and patients with normal placentas. Univariate and multivariated logistic regression analyses were used to evaluate the value of the above parameters for differentiating PAS disorders. Receiver operating characteristics (ROC) curve analyses were used to evaluate the diagnostic efficiency of different diffusion parameters for predicting PAS disorders.

**Results:**

Multivariate analysis demonstrated that only D mean and D max differed significantly among all the studied parameters for differentiating PAS disorders when comparisons between accreta lesions in patients with PAS (AP) and whole placentas in patients with normal placentas (WP-normal) were performed (all *p* < 0.05). For discriminating PAS disorders, a combined use of these two parameters yielded an AUC of 0.93 with sensitivity, specificity, and accuracy of 83.08, 88.89, and 83.70%, respectively.

**Conclusion:**

The diagnostic performance of the parameters from accreta lesions was better than that of the whole placenta. D mean and D max were associated with PAS disorders.

**Supplementary Information:**

The online version contains supplementary material available at 10.1186/s12884-022-04644-9.

## Key points

1. Volumetric analysis of different DWI models can lend strong support to quantitative evaluation of the heterogeneity, cellularity, and microvascular perfusion of the PAS disorders.
2. PAS disorders can be differentiated effectively with the combined use of the different DWI parameters.

## Background

Placenta accreta spectrum (PAS) disorders refer to an abnormal condition where the placental chorionic villi adhere to or invade the myometrium, entering into a place with deficient decidual formation [1, 2]. The incidence of PAS kept rising from 1 in 30,000 to 1 in 300 gravidas over the past four decades [3, 4], mainly as cesarean deliveries increasing [5]. The major problem of PAS resulting from unsuccessful clean placental detachment after delivery is massive obstetric hemorrhage accompanied by secondary complications including coagulopathy, multisystem organ failure, hysterectomy, and even death [6–10].

Ultrasonography remains the first-line modality for detecting PAS disorders but is limited in assessing the posterior placenta and by patients’ body habitus. MRI has been increasingly a complementary modality for the prenatal diagnosis of PAS disorders in recent years. However, according to a recent systematic review, the sensitivity and specificity of MRI in diagnosing PAS varied widely between 75 and 100% and 65–100%, respectively [11]. The discrimination of typical MRI features related to PAS disorders is proposed to require expertise and doctors’ experience. The interobserver agreement in identifying the presence and depth of placental invasion was excellent, but the interrater agreement in ascertaining the topography of the invasion was lower even in hands of experienced examiners [12].

Meanwhile, quantification of placental function and identification of mothers at high risk are both important in today’s clinical practice. Conventional DWI is a monoexponential Gaussian model, evaluating the diffusion restriction of water molecules in tissue using apparent diffusion coefficient (ADC). eADC (exponential apparent diffusion coefficient) is another parameter of DWI used in recent years with eADC images eliminating the T2 shine through effect. However, it is considered that the diffusion of water molecules in biological tissue is much more complicated and restricted by microstructures, such as cell membranes and organelles, which follow a non-Gaussian behavior [13]. Diffusion kurtosis imaging (DKI), first proposed by Jensen et al. [14, 15], allows the accurate estimation of water diffusivity in biological tissue and quantification of tissue heterogeneity and cellularity with higher b values [13, 14]. However, the measured diffusion signals in living tissue are also influenced by the perfusion of blood micro-vascularization at low b values besides the motion of water molecules [16, 17]. IVIM, first proposed by Le Bihan et al. [16], is a biexponential model that separates microvascular perfusion from water diffusivity within the tissue [16, 17]. Some researchers have investigated the feasibility of IVIM in pregnancies complicated by placental dysfunction due to vascular malperfusion [18], placenta accreta [19–21], preeclampsia [22], growth-restricted pregnancy [23], and normal pregnancies [24–26].

Theoretically, PAS placenta might show vascularity and blood flow different from the normal placenta and these changes can be detected by different DWI models, and thus we here attempted to determine whether these DWI models can be used to predict PAS. Therefore, this study primarily aimed to apply these promising advanced DWI models besides the conventional DWI model to evaluate the placental function in patients with PAS disorders and, secondly, evaluate whether the diagnostic parameters derived from different DWI models could be served as quantitative biomarkers for diagnosing PAS disorders.

## Materials and methods

**Fig. 1:**
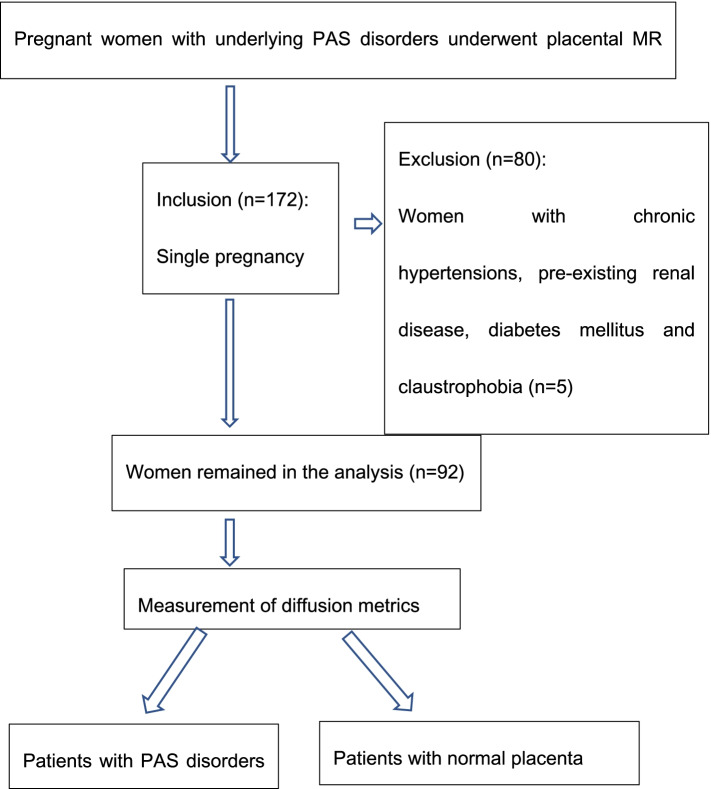
Flowchart of the study design

This study was approved by the institutional review board (IRB) and obtained written informed consent from each woman participants. During November 2018 and March 2021, a total of 172 patients were initially scanned with a DWI sequence during the study period. The inclusion criteria were (1) suspected PAS disorders based on clinical risk factors or previous ultrasound (US) results, (2) singleton pregnancy, (3) fetal development coinciding with gestational age. Patients were excluded for the following reasons (1) chronic hypertension, pre-existing renal disease, and diabetes mellitus, (2) inadequate surgical records, (3) suspected placental insufficiency, (4) severe artifacts on MRI images. Placenta previa without PAS was considered normal in this study. Finally, a total of 92 patients (mean age 31.67 ± 4.58 years, range 22–45 years) were enrolled (Fig. 1). The average gestational age was 32 weeks (range 16–38 weeks).

### MRI protocols

MRI examinations were performed on a 1.5 T MR scanner (Aera, Siemens Healthineers, Erlangen, Germany) using a 16-channel body matrix coil. Conventional MR sequences including HASTE, True-FISP, and T1WI were scanned, then DWI was performed by using a single-shot echo-planar imaging (EPI) sequence with a pair of rectangular diffusion gradient pulses along all three orthogonal axes. The imaging parameters were as follows: TR/TE = 5200/83 ms, number of averages = 2, acquisition matrix = 192 × 120, field of view (FOV) = 390 mm, slice thickness = 5 mm, intersection gap = 5 mm, and parallel imaging acceleration factor = 2. Eleven different b values ranging from 0 to 1600 s/mm^2^ (b = 0, 50, 100, 150, 200, 400, 600, 800, 1000, 1200, and 1600 s/mm^2^) were applied. The total scan time for the DWI sequence was 7 min 29 s.

### Image processing and analysis

ROI delineation and calculation of the DWI, DKI, IVIM parameters were performed using research software IMAgenGINE (Vusion Tech) [27]. For standard monoexponential DWI analysis, b values of 0 and 1000 s/mm^2^ were used to fit the following equation:

$$\mathrm{Sb}/\mathrm S0\:=\:\exp\;(-\mathrm b\times\mathrm{ADC})$$

where Sb and S0 are the signal intensities in the diffusion gradient factors of b and 0, respectively. ADC can be calculated by fitting the signal to this model, and the exponential ADC (eADC) is calculated through the formula:

$$\mathrm{Sb}/\mathrm S0\:=\:\mathrm{Exponential}\;\mathrm{ADC}\:=\:\exp\;\lbrack-(\mathrm b\times\mathrm{ADC})\rbrack$$

For DKI analysis, 6 b-values (b = 0, 400, 800, 1000, 1200, and 1600 s/mm^2^) are used to perform the model fitting the following equation [28, 29]:

$$\mathrm{Sb}/\mathrm S0=\exp\;(-\mathrm b\times\mathrm{MD}+\mathrm b^{2}\cdot\mathrm{MD}^{2}\times\mathrm{MK}/6)$$

where Sb and S0 are the signal intensities acquired with the diffusion gradient factors of b and 0, respectively. MD is the mean diffusivity representing the corrected ADC, MK is the diffusion kurtosis.

For IVIM analysis, 8 b-values (b = 0, 50, 100, 150, 200, 400, 600, 800 s/mm^2^) were used to perform the model fitting the following equation [30, 31]:

$$\mathrm{Sb}/\mathrm S0=(1-\mathrm f)\;\exp\;(-\mathrm b\times\mathrm D)+\mathrm f\;\exp\;\lbrack-\mathrm b\times(\mathrm D+\mathrm D^{\ast})\rbrack$$

where Sb and S0 are the signal intensities in the diffusion gradient factors of b and 0, respectively. IVIM parameters, including D, D*, and f, can be derived from the model. f is the perfusion fraction, D is the diffusion coefficient, and D* is the pseudo-diffusion coefficient.

All ROIs were drawn independently by two radiologists with 5 and 8 years of experience in obstetric imaging, respectively. All patients’ whole placenta ROIs were drawn on each consecutive DWI with b = 0 s/mm^2^ covering the whole placenta, referring to conventional T2WI. PAS-ROIs were drawn covering the accreta lesions. The ROI size was slightly smaller than the visible margin of the placenta to avoid partial volume effect and was then automatically copied to all diffusion parameter maps. The mean, minimum, and maximum ADC (ADC mean, ADC min, and ADC max), eADC (eADC mean, eADC min, and eADC max), MD (MD mean, MD min, and MD max), MK (MK mean, MK min, and MK max), D (D mean, D min, and D max), D* (D* mean, D* min, and D* max), and f (f mean, f min, and f max) values were automatically calculated, and the diffusion parameter maps were also automatically produced. The measurements made by the first reader were used to evaluate the intra-reader reproducibility with a minimum washout period of at least 1 month, while the measurements made by two readers were used to evaluate the inter-reader reproducibility and were averaged for statistical analysis.

### Reference standard

The diagnosis of PAS disorders was made intraoperatively. Placenta percreta was diagnosed when the placental tissue invaded the uterine serosa and surrounding organs, including the broad ligament, vaginal wall, and bladder visually [32]. During the 3rd stage of labor, placenta increta was diagnosed when the placenta did not separate after 20 min despite active management, resulting in a difficult manual piecemeal removal of the placenta and heavy bleeding from the implantation site [32]. Placenta creta was diagnosed when the placenta firmly adhered to the endometrium with uncontrollable bleeding at the time of removal [32]. Pathological examination was performed from uterine specimens in hysterectomy cases or from placental tissue in the invasive site.

### Statistical analysis

The heterogeneity and diffusion parameters of the placenta changed with the increase of gestational weeks. All the parameters were corrected using the following equation to remove changes induced by gestational age [33]:

$${y}_{corrected}=y-\boldsymbol{\beta} {\boldsymbol{X}}_{\boldsymbol{age}}=y-\left(\begin{array}{c}{\beta}_0\\ {}{\beta}_c\end{array}\right){\boldsymbol{X}}_{\boldsymbol{age}}$$

Where *y*_*corrected*_ is the value after correction, *y* is the original value, ***X***_***age***_ is the gestational week vector, and *β*_0_ and *β*_*c*_ are the constant and the linear regression coefficient in linear fitting, respectively.

A two-sample independent t-test was used to compare the difference in the DWI parameters of the whole placenta between patients with PAS disorders and patients with normal placenta, and also was used to compare the difference between accreta lesions in patients with PAS disorders and patients with normal placentas. Receiver operating characteristics (ROC) analyses were performed to evaluate the diagnostic performance of significant parameters from measuring the whole placenta and accreta lesions in patients with PAS disorders, respectively, in predicting PAS disorders. Youden index and corresponding sensitivity, specificity, positive and negative likelihood ratio were calculated. Z test was used to compare AUCs. The significant DWI parameters showing the highest Youden index were included for the differentiation. Univariate and multivariated logistic regression analyses were used to identify independent risk factors of PAS disorders. The inter-reader and intra-reader reproducibility for parameter measurements was evaluated using the intraclass correlation coefficient (ICC) with 95% confidence intervals (CI). *P* values < 0.05 were considered statistically significant. All analyses were performed on a Win10 desktop with Python 3.6, scipy 1.5.2 and sklearn 0.24.1 [34].

## Results

Table 1 presents the maternal characteristics of the study participants. Sixty-five patients (70.65%)with PAS disorders including 12 (13.04%)patients with placenta creta, 46(50%) patients with placenta increta, 7(7.61%)patients with placenta percreta, and 27(29.35%) patients with normal placentas remained in the analysis. Of the 27 patients without PAS disorders,16 patients had vaginal delivery and 11 patients had cesarean delivery. Of the 65 patients with PAS disorders, 3 patients had hysterectomy, 57 patients had forcible separation and hemostasis, 5 patients had forcible separation without hemostasis.

**Table 1:** Maternal characteristics in the study groups

	Patients with normal placenta	Patients with PAS disorders	*P* value
**Number**	27	65	
**Age (years)**	29.37 ± 4.34	32.63 ± 4.36	0.026
Less than 35	24 (88.89%)	43 (66.15%)	
35 or older	3 (11.11%)	22 (33.85%)	
**Gestational age** **At examination (weeks)**	32 (6)	32 (5)	0.611
**Gestational age** **At the time of delivery (weeks)**	38 (2)	36 (3)	0.000
**Number of Previous caesarean Section**			
0	13 (48.15%)	7 (10.77%)	
1	12 (44.44%)	54 (83.08%)	
2 or more	2 (7.41%)	4 (6.15%)	0.000
**Previous uterine Dilation and Curettage**			
No	9 (3.33%)	2 (3.01%)	
Yes	18 (66.67%)	63 (96.92%)	0.000
**Placenta previa**			0.000
**No**	16 (59.26%)	4 (6.15%)	
**Yes**	11 (40.74%)	61 (93.85%)	
**Placental position**			0.052
**Anterior**	12 (44.44%)	30 (46.15%)	
**Posterior**	12 (44.44%)	15 (23.08%)	
**Anterior + posterior**	3 (11.11%)	20 (30.77%)	

Patients with PAS disorders were older than those with normal placentas (*p* = 0.002), and more patients with PAS disorders were over 35 years old (*p* = 0.026). Patients with PAS disorders also delivered earlier, had more CDs, previous uterine dilations and curettages, and placenta previa (*p* = 0.000 respectively).

Intra- and inter-observer agreement varied from 0.839 to 0.979 (Table 2). Overall, the Intra- and inter-observer agreements were excellent for the volumetric analysis of the placenta.

**Table 2:** The inter-reader and intra-reader reproducibility for DWI parameters

parameters	ICC (95% CI)	
	inter-reader	intra-reader
**Standard DWI parameters**		
ADC mean (×10^−3^ mm^2^/s)	0.839 (0.721–0.906)	0.978 (0.954–0.990)
eADC mean	0.980 (0.958–0.991)	0.975 (0.945–0.988)
**DKI parameters**		
MD mean (×10^−3^ mm^2^/s)	0.927 (0.851–0.965)	0.917 (0.833–0.960)
MK mean	0.979 (0.956–0.990)	0.986 (0.972–0.994)
**IVIM parameters**		
f mean (%)	0.914 (0.824–0.958)	0.904 (0.809–0.953)
D mean (×10^−3^ mm^2^/s)	0.968 (0.935–0.985))	0.976 (0.951–0.989))
D* mean (×10^−3^ mm^2^/s)	0.972 (0.942–0.987)	0.915(0.828–0.958)

**Fig. 2:**
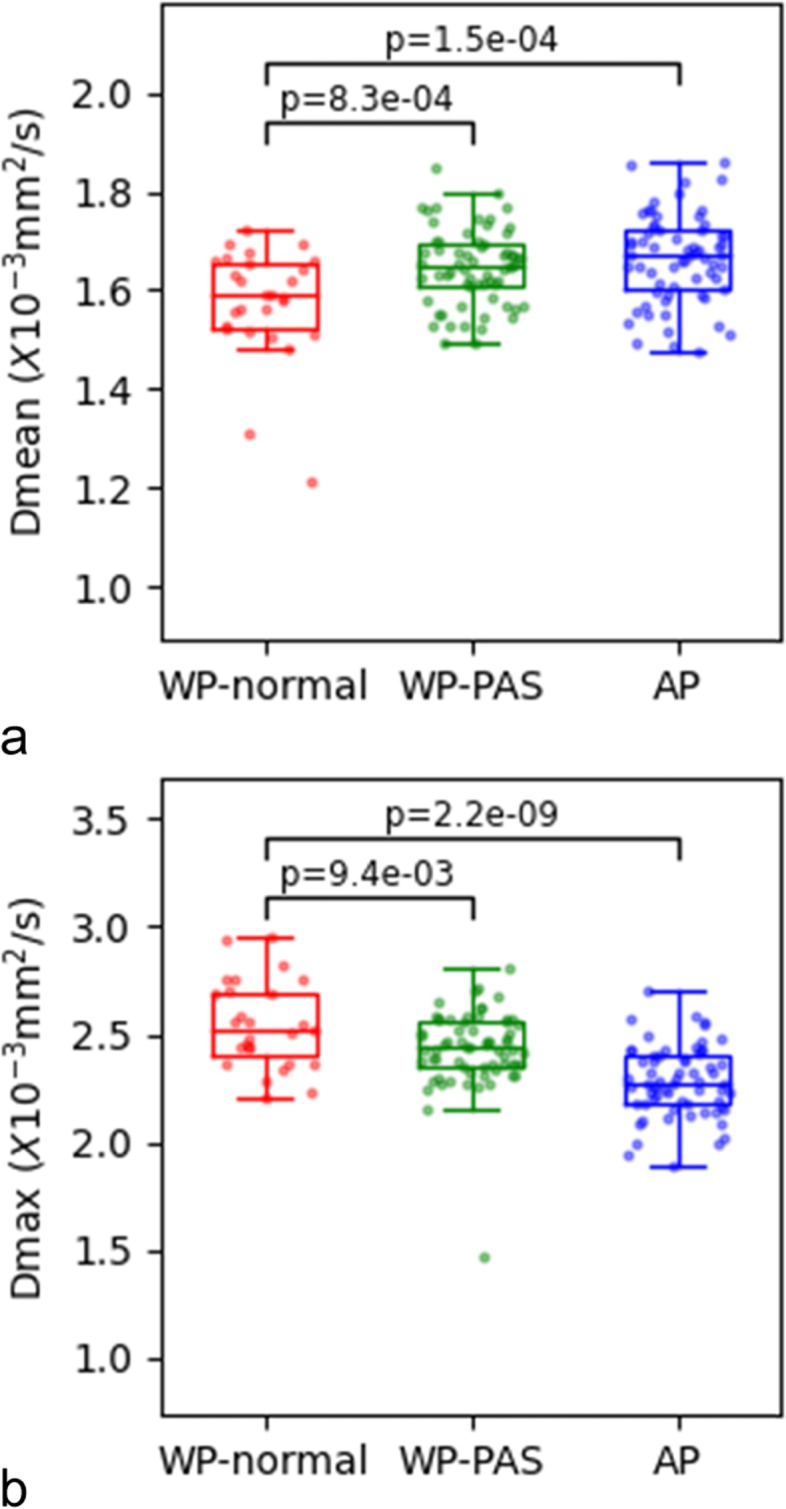
Box and whisker plots of D mean and D max for patients with normal placentas (Whole placenta-normal), patients with PAS disorders (Whole placenta-PAS) and accreta lesions in patients with PAS (AP)

**Fig. 3:**
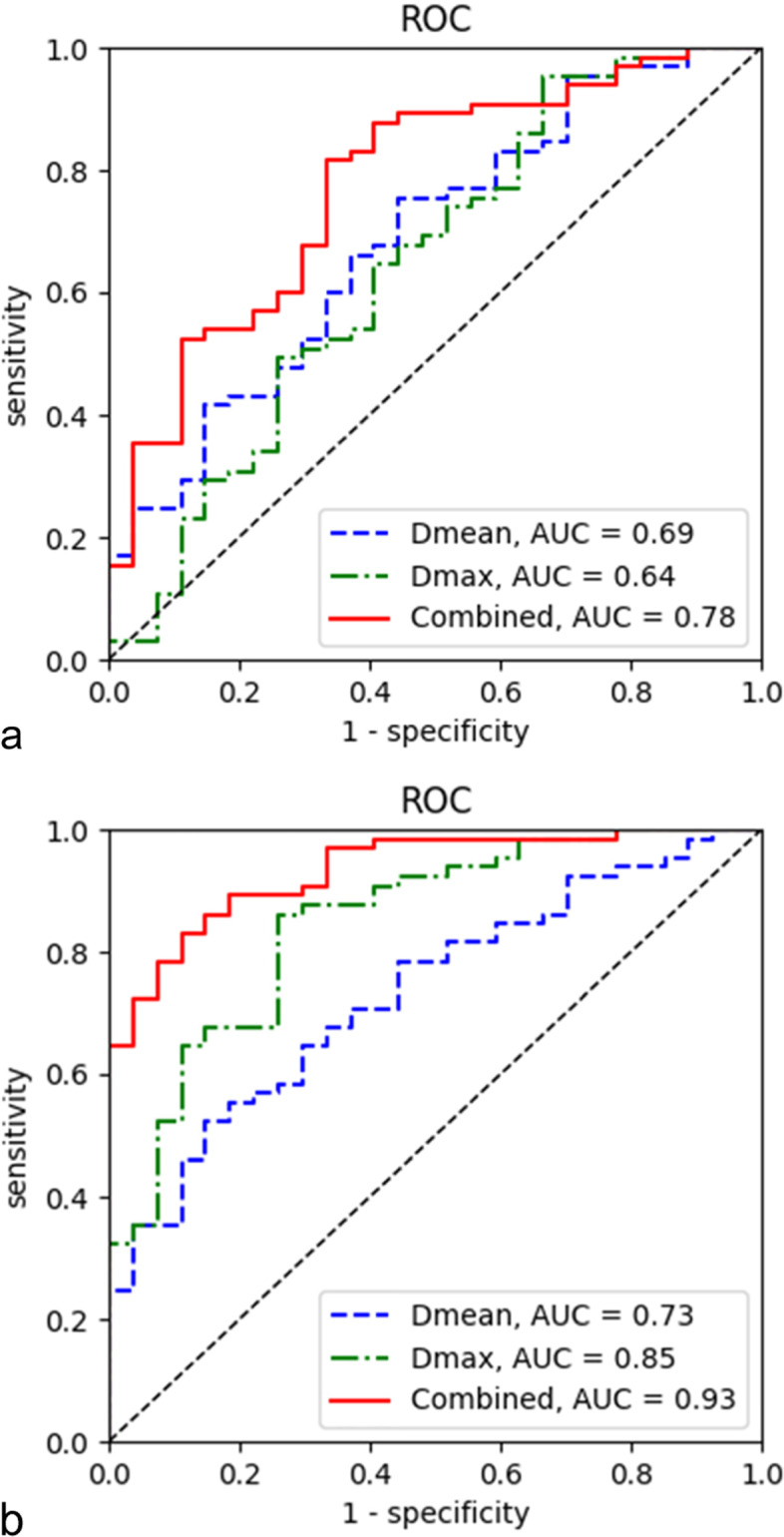
ROC (Receiver operating characteristics) curves for predicting PAS disorders based on parameters from different DWI (diffusion weighted imaging) models. **a** parameters from whole placenta comparisons, **b** parameters from accreta lesions vs normal placentas

Volumetric analysis of whole placenta comparisons between patients with normal placentas (WP-normal) and patients with PAS disorders (WP-PAS) was performed, showing that MD mean, MD max, f mean, D mean, and D* mean were significantly higher while MK mean, MD min and D max were significantly lower in patients with PAS disorders (all *p* < 0.05) (Fig. 2, Table 3, supplement 1). At multivariate analysis, D mean (OR:2.45, 95% CI, 1.37–4.4) and D max (OR:0.45, 95% CI, 0.25–0.82) were identified as independent risk factors for PAS disorders. For discriminating PAS disorders, the AUCs of the above 2 parameters were 0.69 and 0.64 respectively while the AUC yielded 0.78 with the combined 2 parameters (Table 4). Significant differences were found in the AUCs between the combination of the 2 parameters and the separate 2 parameters (all *p* < 0.05) (Fig. 3).

**Table 3:** Comparison of DWI parameters between patients with normal placenta and patients with PAS disorders (*n* = 92)

parameters	Patients with PAS disorders		Patients with normal placenta	*P* value	
	Whole placenta	Accreta lesions	Whole placenta	WP-normal vs WP-PAS	AP vs WP-normal
**Standard DWI parameters**					
ADC mean (×10^− 3^ mm^2^/s)	1.55 (0.08)	1.56 (0.09)	1.51 (0.12)	0.076	0.180
ADC min (×10^−3^ mm^2^/s)	0.27 (0.39)	0.51 (0.46)	0.40 (0.32)	0.061	0.265
ADCmax (×10^−3^ mm^2^/s)	2.43 (0.24)	2.29 (0.24)	2.49 (0.26)	0.094	0.000
eADC mean	0.09 (0.01)	0.09 (0.01)	0.10 (0.02)	0.099	0.155
eADC min	0.01 (0.01)	0.02 (0.01)	0.01 (0.01)	0.394	0.055
eADC max	0.67 (0.32)	0.46 (0.38)	0.53 (0.29)	0.096	0.522
**DKI parameters**					
MD mean (×10^−3^ mm^2^/s)	3.17 (0.39)	3.15 (0.47)	2.92 (0.26)	0.005	0.005
MD min (×10^− 3^ mm^2^/s)	0.26 (0.56)	0.71 (0.84)	0.44 (0.76)	0.034	0.21
MD max (×10^−3^ mm^2^/s)	7.41 (0.92)	7.14 (1.19)	7.12 (0.56)	0.000	0.29
MK mean	0.52(0.04)	0.52 (0.04)	0.53 (0.04)	0.032	0.041
MK min	0 (0)	0 (0)	0 (0)	N/A	N/A
MK max	1.68 (1.50)	1.23 (0.75)	1.87 (1.52)	0.47	0.032
**IVIM parameters**					
f mean (%)	43.89 (4.65)	43.18 (8.36)	41.59 (4.50)	0.010	0.041
f min (%)	0 (0)	0 (0)	0 (0)	N/A	N/A
f max (%)	100 (0)	100 (0)	100 (0)	N/A	N/A
D mean (×10^−3^ mm^2^/s)	1.65 (0.08)	1.67 (0.12)	1.59 (0.13)	0.000	0.000
D min (×10^−3^ mm^2^/s)	0.31 (0.48)	0.54 (0.53)	0.37 (0.50)	0.067	0.186
D max (×10^−3^ mm^2^/s)	2.44 (0.21)	2.27 (0.22)	2.52 (0.29)	0.000	0.000
D* mean (×10^−3^ mm^2^/s)	36.0 (9.04)	38.31 (11.18)	30.34 (11.58)	0.012	0.000
D* min (×10^−3^ mm^2^/s)	0 (0.02)	0.29 (0.65)	0 (0)	N/A	N/A
D* max (×10^−3^ mm^2^/s)	100 (0)	100 (0)	100 (0)	N/A	N/A

**Table 4:** Diagnostic value of DWI parameters in differentiating PAS disorders

group	AUC(95%CI)	Optimal cutoff value	Youden index	sensitivity	specificity	+LR	-LR
WP-normal vs WP-PAS							
f mean(%)	0.67 (0.56, 0.79)	44	0.36	50.77%	85.19%	3.43	0.58
D mean(∙10^−3^ mm^2^/s)	0.69 (0.58, 0.80)	1.61	0.31	75.38%	55.56%	1.70	0.44
D max(∙10^−3^ mm^2^/s)	0.64 (0.52, 0.76)	1.75	0.29	95.38%	33.33%	1.43	0.14
D* mean(∙10^−3^ mm^2^/s)	0.68 (0.57, 0.79)	27.29	0.34	96.92%	37.04%	1.54	0.08
MD mean(×10^−3^ mm^2^/s)	0.68 (0.57,0.79)	3.11	0.37	55.38%	81.48%	2.99	0.55
MD min(×10^−3^ mm^2^/s)	0.63 (0.51, 0.75)	0.50	0.24	83.08%	40.74%	1.40	0.42
MD max(×10^−3^ mm^2^/s)	0.71 (0.60, 0.82)	7.22	0.49	63.08%	77.78%	2.84	0.48
Mkmean	0.63 (0.51, 0.75)	0.64	0.28	53.85%	74.07%	2.08	0.62
Combined (D mean and D max)	0.78 (0.68, 0.87)	0.46	0.48	81.54%	66.67%	2.45	0.28
AP vs WP-normal							
ADC max(∙10^−3^ mm^2^/s)	0.83 (0.75, 0.91)	2.55	0.57	83.08%	74.07%	3.20	0.23
f mean(%)	0.62 (0.50 0.74)	44	0.34	49.23%	85.19%	3.32	0.60
D mean(∙10^−3^ mm^2^/s)	0.73 (0.63, 0.84)	1.67	0.38	52.31%	85.19%	3.53	0.56
D max(∙10^−3^ mm^2^/s)	0.85 (0.77, 0.93)	2.41	0.60	86.15%	74.07%	3.32	0.19
D* mean(∙10^−3^ mm^2^/s)	0.75 (0.65, 0.85)	30.38	0.41	89.23%	51.85%	1.85	0.21
MD mean(×10^−3^ mm^2^/s)	0.68 (0.57, 0.79)	3.12	0.37	55.38%	81.485	2.99	0.55
Mkmean	0.63 (0.51, 0.75)	0.64	0.26	52.31%	74.07%	2.02	0.64
Mkmax	0.64 (0.52, 0.76)	1.52	0.30	75.38%	55.56%	1.70	0.44
Combined (D mean and D max)	0.93 (0.89, 0.98)	0.46	0.72	83.08%	88.89%	7.48	0.19

**Fig. 4:**
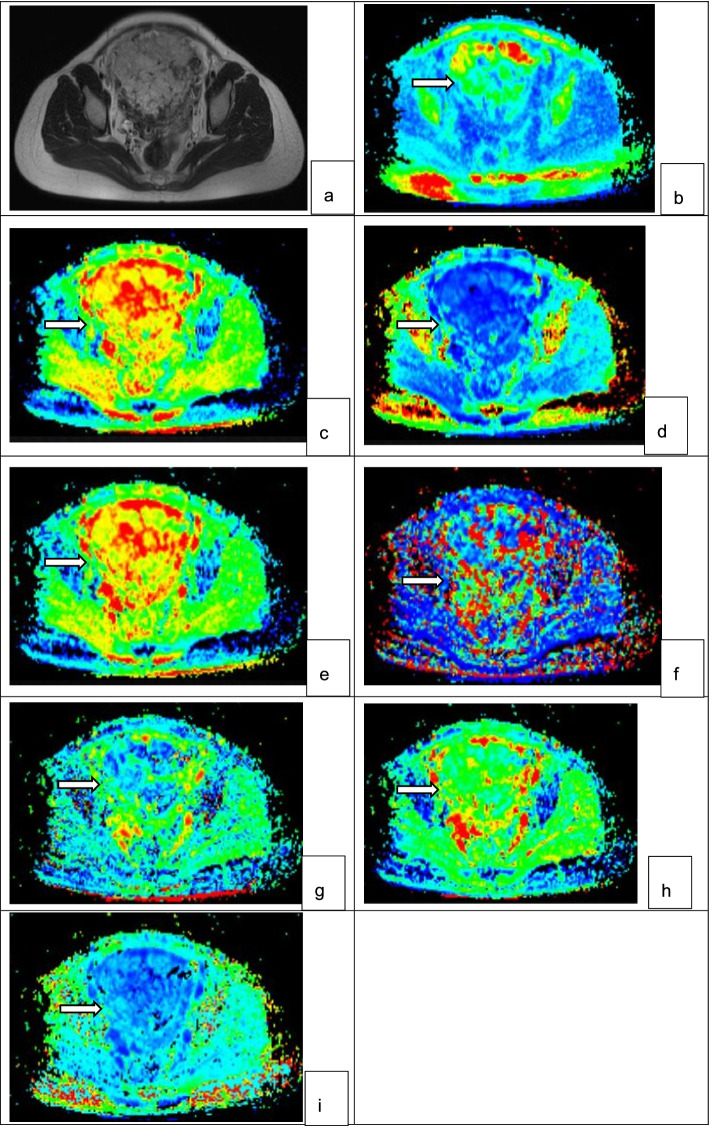
PAS disorders in a 33-year-old woman with prior cesarean delivery and placenta previa. (**a**)T2WI(T2 weighted imaging) showed abnormal vascularization of placental bed and abnormal intraplacental vascularity. The accreta lesion was generally iso- to hyperintense on DWI (**b**), heterogeneously hyperintense on ADC map (**c**) and hyperintense on eADC map (**d**) (white arrow). On IVIM(intravoxel incoherent motion) images, the lesions appeared heterogeneously hyperintense on D map (**e**), heterogeneous on D* map (**f**) and hypo- to isointense with some areas of hyperintense on f map (**g**) (white arrow). On DKI (diffusion kurtosis imaging) images, the lesions appeared iso- to hyperintense on MD map (**h**) and hypointense on MK map (**i**) (white arrow)

Secondly, comparisons between accreta lesions in patients with PAS (AP) and whole placentas in patients with normal placentas (WP-normal) using volumetric analysis were performed. It demonstrated that MD mean, f mean, D mean and D* mean were significantly higher while ADC max, MK mean, MK max and D max were significantly lower in patients with PAS disorders (all *p* < 0.05) (Figs. 2 and 4, Table 3, supplement 1). At multivariate analysis, D mean (OR:4.12, 95% CI, 1.85–9.18) and D max (OR:0.15, 95% CI, 0.07–0.34) were identified as independent risk factors for PAS disorders. For discriminating PAS disorders, the AUCs of the above 2 parameters were 0.73 and 0.85 respectively while the AUC yielded 0.93 with the combined use of the 2 significant parameters (Table 4). Significant differences were found in the AUCs between the combination of the 2 parameters and D mean and D max (all *p* < 0.05) (Fig. 3).

## Discussion

Traditionally, most of the prior investigations [18–22, 24, 35, 36] measured DWI parameters from manually placed ROIs on one or several representative slices of the placenta, which may lead to interobserver variability in ROI selection. In addition, inappropriate ROI selection may not accurately reflect the physiological features of the placenta. Entire lesion volumetric analysis was adopted in our study to capture the parameters of the entire placenta and the accreta lesions in PAS disorders to eliminate sampling bias during data processing potentially. The intra- and interobserver agreements were excellent for all the parameters using the volumetric analysis. Therefore, the VOI-based estimation of DWI parameters was highly reproducible and repeatable.

Some studies have adopted IVIM to evaluate the placental function. Capuani’s study demonstrated that ADC decreased with GA increase while f increased [24]. Jakab’s study also demonstrated that f was moderately increasing during gestation [25]. However, Derwig’s study reported that f did not change with GA [36], Sohlberg’s and Kristi B’s study reported that f decreased with GA [18, 22]. The inconsistency of the results from the above researches may arise from different ROI selections and different IVIM protocols. As for the influence of GA, parameters corrections were performed before the analysis to demonstrate the difference of the parameters between normal placentas and PAS disorders.

The placenta is a highly vascular organ containing both maternal and fetal vascular systems appropriate for IVIM analysis. The IVIM technique separates placental diffusion and perfusion based on the hypothesis that the diffusion-weighted signal in a voxel depends on a perfusion compartment described by D* and f and a diffusion compartment described by D. D is governed by random Brownian motion, representing the physical characteristic of the tissue, like cell size and membrane permeability.

Our study showed that D mean and D max were independent risk factor for PAS disorders from comparisons between accreta lesions in patients with PAS (AP) and whole placentas in patients with normal placentas. The current hypothesis is supposed that the endometrium-myometrial interface defect caused by uterine scar leads to a failure of normal decidualization and allows extravillous trophoblastic infiltration and villous development in the deep myometrium [37]. Capuani’s study identified that the normal placenta had higher cellularity and abundant cytoplasm, hindering water diffusion [24]. In this study, the D mean was significantly lower in normal placentas than in accreta lesions, supporting the previous hypothesis. D max was probably measured in areas with less water diffusion restriction. It had higher diagnostic accuracy than D mean in the accreta lesions, suggesting the noteworthy increased cellularity in certain areas in the accreta lesions. The diagnostic performance of the parameters from accreta lesions was better than parameters from the whole placenta. We further combined D mean and D max for predicting PAS disorders, the diagnostic performance improved significantly with higher sensitivity, specificity, and accuracy of 83.08, 88.89, and 93.39%, respectively. It suggested the feasible combined use of the 2 DWI parameters in the quantitative evaluation and prediction of PAS disorders.

In the placenta, f represents the moving blood volume fraction compared with the total voxel volume and D* represents the blood movement in the intervillous spaces and the fetal capillaries [26]. In patients with PAS disorders, Uterine scars from previous CDs caused the local decidual deficit and the increasing number of partially or non-remodeled spiral arteries with abnormal EVT invasion into radial and arcuate arteries deep within the myometrium. It resulted in a hypervascular placental bed and massive blood loss when the invasive placenta detached [37, 38]. So the perfusion fraction increased in the accreta lesions and finally involved the whole placenta in patients with PAS disorders. Maternal blood flows slowly through the large pools of IVS, bathing the fetal villi and enabling oxygen exchange between mothers and fetuses. The elevated D* mean values reflected the increased blood movement in the IVS and the fetal capillaries in the accreta lesions.

DKI has been increasingly implemented for providing more precise information on tissue cellularity and heterogeneity than conventional DWI. Because the former can quantify the non-Gaussian behavior of water diffusion, which is believed to reveal more genuine water molecular movement and distribution in biological tissues. MD is a corrected ADC value for the non-Gaussian diffusion and a diffusion-related coefficient. MD is correlated with tissue cellularity, showing a similar change to D in this study as MD mean values are significantly higher in accreta lesions. MK from DKI reflects the complexity or heterogeneity of the tissue. Thus the more significantly higher MK mean and MK max values represent more heterogeneous and irregular tissue components in normal placentas.

This study took the lead in combining two promising functional DWI models, DKI, and IVIM with conventional DWI models to characterize placental heterogeneity, cellularity, and microvascular perfusion at the same time and to distinguish PAS disorders. In this study, the accreta lesions were hyperperfused with increased blood movement in the IVS and the fetal capillaries but with deceased cellularity and heterogeneity compared with normal placentas. These changes were prominent enough to involve the whole placenta but were more profound in the accreta lesions.

This study had some limitations. First, this study is retrospective with a small sample size, with the selection bias inevitable. Second, It was impossible to obtain breath-hold imaging from pregnant women, so the free-breathing protocol in this study was utilized. The good reproducibility of results confirmed the reliability of the measurements. Third, this study utilized 1600 s/mm^2^ as the maximum b value, which is smaller than the recommended 2000 s/mm^2^ for the rectal, renal, and hepatic lesions [29, 30, 39]. In this study, 11 b values ranging 0–1600 s/mm^2^ in 3 orthogonal directions were adopted in the DKI sequence. It was feasible to use this DKI model in placental imaging because the DKI protocol in this study showed satisfactory overall imaging quality to the perfect intra- and inter-observer agreements.

In conclusion, placental function in PAS disorders changed miscellaneously, involving diffusion, perfusion, and heterogeneity of the placenta. Abnormal decidualization and subsequent villous infiltration in the myometrium resulted in the deceased cellularity and heterogeneity in the accreta lesions in adjunction with the increased relative amount of blood flowing through the vascular bed. Therefore, it is worthwhile and necessary to combine different DWI parameters in the comprehensive evaluation and accurate diagnosis of PAS disorders. Secondly, PAS disorders can be differentiated with the combined use of D mean and D max.

## Supplementary Information

**Additional file 1.** Supplementary Figure Box and whisker plots of ADC (ADC mean, ADC min, and ADC max), eADC (eADC mean, eADC min, and eADC max), MD (MD mean, MD min, and MD max), MK (MK mean and MK max), D (D min), D* (D* mean), and f (f mean) for patients with normal placentas (Whole placenta-normal), patients with PAS disorders (Whole placenta-PAS) and accreta lesions in patients with PAS (AP).

## Data Availability

The datasets generated during and analyzed during the current study are not publicly available due to PACS system regulated by Sichuan Provincial People’s Hospital, but are available from the corresponding author on reasonable request.

## Abbreviations

MRI: Magnetic resonance imaging
PAS: Placenta accreta spectrum
DWI: Diffusion weighted imaging
IVIM: Intravoxel incoherent motion
DKI: Diffusion kurtosis imaging
ADC: Apparent diffusion coefficient
eADC: Exponential apparent diffusion coefficient
MK: Mean kurtosis
MD: Mean diffusion coefficient
ROC: Receiver operating characteristics
WP-normal: Whole placentas in patients with normal placenta
WP-PAS: Whole placentas in patients with PAS
AP: Accreta lesions in patients with PAS
US: Ultrasonography
SAR: Society of Abdominal Radiology
ESUR: European Society of Urogenital Radiology
ROI: Region of interest
GA: Gestational age
CD: Cesarean delivery
ME: Monoexponential
