# Supplementary figures and images for: Monoexponential, biexponential and diffusion kurtosis MR imaging models: quantitative biomarkers in the diagnosis of placenta accreta spectrum disorders
Source: BMC Pregnancy Childbirth. 2022 Apr 22;22:349. doi: 10.1186/s12884-022-04644-9 (PMC9034554; doi:10.1186/s12884-022-04644-9)

| 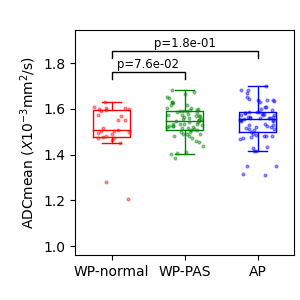 | 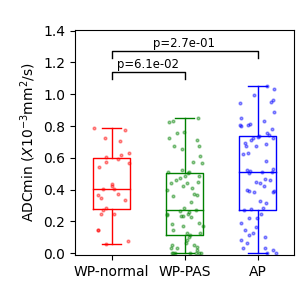 |
| --- | --- |
| 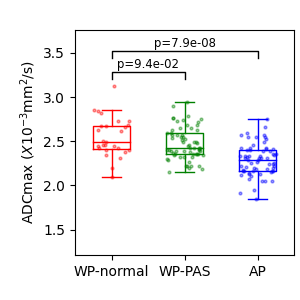 | 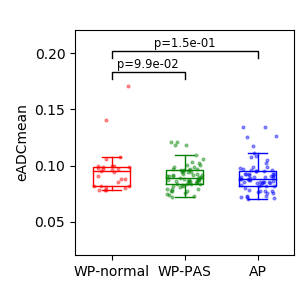 |
| 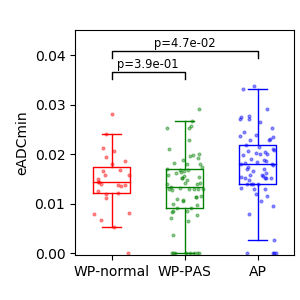 | 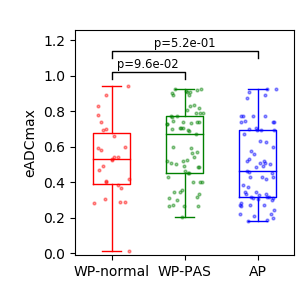 |
| 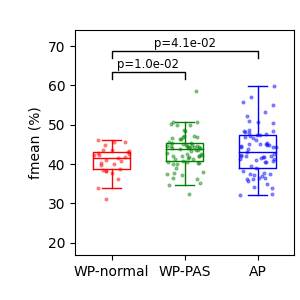 | 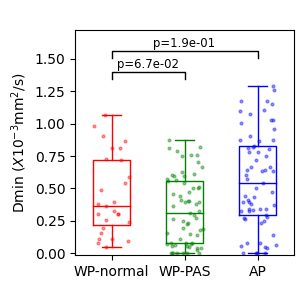 |
| 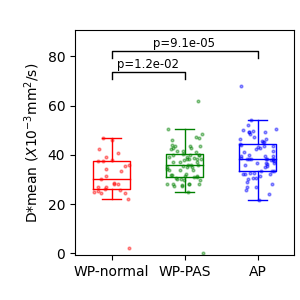 | 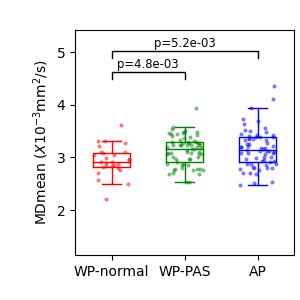 |
| 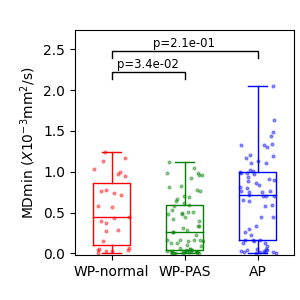 | 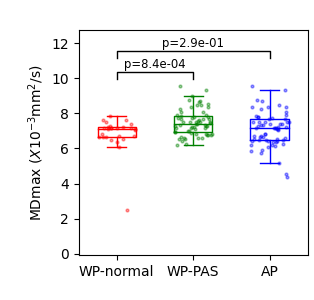 |
| 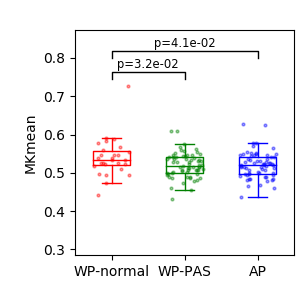 | 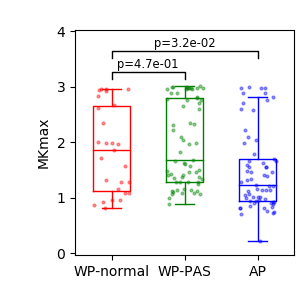 |

Supplement: Supplementary file 1 — Additional file 1. Supplementary Figure Box and whisker plots of ADC (ADC mean, ADC min, and ADC max), eADC (eADC mean, eADC min, and eADC max), MD (MD mean, MD min, and MD max), MK (MK mean and MK max), D (D min), D* (D* mean), and f (f mean) for patients with normal placentas (Whole placenta-normal), patients with PAS disorders (Whole placenta-PAS) and accreta lesions in patients with PAS (AP). [file 12884_2022_4644_MOESM1_ESM.docx]
